# Supplementary material for: Resolving the full spectrum of human genome variation using Linked-Reads
Source: Genome Res. 2019 Apr;29(4):635–45. doi: 10.1101/gr.234443.118 (PMC6442396; doi:10.1101/gr.234443.118)
Supplement: Supplemental Material [file supp_gr.234443.118_Supplemental_longranger-2.2.zip › longranger-2.2.tar.bz2!/lr2.2-public/lib/stan/cmdstan/stan/doc/api/html/append__col_8hpp_source.html]

Stan: src/stan/math/matrix/append\_col.hpp Source File


|  |  |
| --- | --- |
|  | Stan  2.5.0  probability, sampling & optimization |


- Main Page
- Related Pages
- Namespaces
- Classes
- Files

- File List
- File Members

All Classes Namespaces Files Functions Variables Typedefs Enumerations Enumerator Friends Macros Pages

- src
- stan
- math
- matrix

append\_col.hpp

Go to the documentation of this file.

1 #ifndef STAN\_\_MATH\_\_MATRIX\_\_APPEND\_\_COL\_HPP

2 #define STAN\_\_MATH\_\_MATRIX\_\_APPEND\_\_COL\_HPP

3

4 #include <stan/math/matrix/Eigen.hpp>

5 #include <stan/meta/traits.hpp> //stan::return\_type

6 #include <stan/math/error\_handling/matrix/check\_size\_match.hpp>

7 #include <vector>

8

9 namespace stan {

10  namespace math {

11

12  using Eigen::Dynamic;

13  using Eigen::Matrix;

14  using std::vector;

15  using stan::math::check\_size\_match;

16

17  //matrix append\_col(matrix, matrix)

18  //matrix append\_col(matrix, vector)

19  //matrix append\_col(vector, matrix)

20  //matrix append\_col(vector, vector)

21  template <typename T1, typename T2, int R1, int C1, int R2, int C2>

22  inline Matrix<typename return\_type<T1, T2>::type, Dynamic, Dynamic>

23  append\_col(const Matrix<T1, R1, C1> & A,

24  const Matrix<T2, R2, C2> & B) {

25  int Arows = A.rows();

26  int Brows = B.rows();

27  int Acols = A.cols();

28  int Bcols = B.cols();

29  check\_size\_match("append\_col(%1%)",

30  Arows, "rows of A",

31  Brows, "rows of B",

32  (double\*)0);

33

34  Matrix<typename return\_type<T1, T2>::type, Dynamic, Dynamic>

35  result(Arows, Acols+Bcols);

36  for (int j = 0; j < Acols; j++)

37  for (int i = 0; i < Arows; i++)

38  result(i, j) = A(i, j);

39

40  for (int j = Acols, k = 0; k < Bcols; j++, k++)

41  for (int i = 0; i < Arows; i++)

42  result(i, j) = B(i, k);

43  return result;

44  }

45

46  //row\_vector append\_col(row\_vector, row\_vector)

47  template <typename T1, typename T2, int C1, int C2>

48  inline Matrix<typename return\_type<T1, T2>::type, 1, Dynamic>

49  append\_col(const Matrix<T1, 1, C1> & A,

50  const Matrix<T2, 1, C2> & B) {

51  int Asize = A.size();

52  int Bsize = B.size();

53  Matrix<typename return\_type<T1, T2>::type, 1, Dynamic>

54  result(Asize + Bsize);

55  for (int i = 0; i < Asize; i++)

56  result(i) = A(i);

57  for (int i = 0, j = Asize; i < Bsize; i++, j++)

58  result(j) = B(i);

59  return result;

60  }

61

62

63  //matrix append\_col(matrix, matrix)

64  //matrix append\_col(matrix, vector)

65  //matrix append\_col(vector, matrix)

66  //matrix append\_col(vector, vector)

67  template <typename T, int R1, int C1, int R2, int C2>

68  inline Matrix<T, Dynamic, Dynamic>

69  append\_col(const Matrix<T, R1, C1> & A,

70  const Matrix<T, R2, C2> & B) {

71  check\_size\_match("append\_col(%1%)",

72  A.rows(), "rows of A",

73  B.rows(), "rows of B",

74  (double\*)0);

75

76  Matrix<T, Dynamic, Dynamic>

77  result(A.rows(), A.cols()+B.cols());

78  result << A, B;

79  return result;

80  }

81

82  //row\_vector append\_col(row\_vector, row\_vector)

83  template <typename T, int C1, int C2>

84  inline Matrix<T, 1, Dynamic>

85  append\_col(const Matrix<T, 1, C1> & A,

86  const Matrix<T, 1, C2> & B) {

87  Matrix<T, 1, Dynamic>

88  result(A.size()+B.size());

89  result << A, B;

90  return result;

91  }

92

93  }

94 }

95 #endif

check\_size\_match.hpp

stan::math::check\_size\_match

bool check\_size\_match(const char \*function, T\_size1 i, const char \*name\_i, T\_size2 j, const char \*name\_j, T\_result \*result)

**Definition:** check\_size\_match.hpp:13

traits.hpp

Eigen.hpp

stan::math::append\_col

Matrix< typename return\_type< T1, T2 >::type, Dynamic, Dynamic > append\_col(const Matrix< T1, R1, C1 > &A, const Matrix< T2, R2, C2 > &B)

**Definition:** append\_col.hpp:23


---

[ Stan Home Page ]

*© 2011–2014,
Stan Development Team.*
